# How to Design a Genetic Mating Scheme: A Basic Training Package for *Drosophila* Genetics

**DOI:** 10.1534/g3.112.004820

**Published:** 2013-02-01

**Authors:** John Roote, Andreas Prokop

**Affiliations:** * Department of Genetics, University of Cambridge, Cambridge CB2 3EH, United Kingdom; † Faculty of Life Sciences, Wellcome Trust Centre for Cell-Matrix Research, M13 9PT, United Kingdom

**Keywords:** Drosophila, classical genetics, transgenesis, education, model organism

## Abstract

*Drosophila melanogaster* is a powerful model organism for biological research. The essential and common instrument of fly research is genetics, the art of applying Mendelian rules in the specific context of *Drosophila* with its unique classical genetic tools and the breadth of modern genetic tools and strategies brought in by molecular biology, transgenic technologies and the use of recombinases. Training newcomers to fly genetics is a complex and time-consuming task but too important to be left to chance. Surprisingly, suitable training resources for beginners currently are not available. Here we provide a training package for basic *Drosophila* genetics, designed to ensure that basic knowledge on all key areas is covered while reducing the time invested by trainers. First, a manual introduces to fly history, rationale for mating schemes, fly handling, Mendelian rules in fly, markers and balancers, mating scheme design, and transgenic technologies. Its self-study is followed by a practical training session on gender and marker selection, introducing real flies under the dissecting microscope. Next, through self-study of a PowerPoint presentation, trainees are guided step-by-step through a mating scheme. Finally, to consolidate knowledge, trainees are asked to design similar mating schemes reflecting routine tasks in a fly laboratory. This exercise requires individual feedback but also provides unique opportunities for trainers to spot weaknesses and strengths of each trainee and take remedial action. This training package is being successfully applied at the Manchester fly facility and may serve as a model for further training resources covering other aspects of fly research.

For a century, the fruit fly *Drosophila* has been used as a powerful model organism for biological research (Ashburner 1993; Bellen *et al.* 2010; Martinez Arias 2008). Initially the fly was the essential vehicle for classical genetics until the basic genetic rules and tools generated during the first half of the 20th century were recognized and used as a powerful means to dissect biological problems (Keller 1996). For the last 50 years, fly genetics has been systematically and successfully applied to decipher principle mechanisms underpinning numerous fundamental biological processes, including development (Lawrence 1992), signaling (Cadigan and Peifer 2009), cell cycle (Lee and Orr-Weaver 2003), nervous system development, function and behavior (Bellen *et al.* 2010; Weiner 1999), and even the molecular aspects of human disease (Bier 2005). Given the high degree of evolutionary conservation, this work has laid important foundations for research in mammals (Bellen *et al.* 2010), and the fly continues to play this role. Its future importance is obvious, for example, when considering the increasing amounts of human disease genes that are being discovered, for many of which the principal biological functions still need to be unraveled.

To carry out such work, classical genetic tools and rules still have a pivotal place in current *Drosophila* research. In addition, fly genetics has been further revolutionized through the advent of molecular biology, the sequencing of the fly genome, the discovery of transposable elements as a vehicle for transgenesis, targeted gene expression systems, the systematic generation of deletions, transposable element insertions and transgenic knock-down constructs covering virtually every *Drosophila* gene, as well as the application of recombinases or target-specific nucleases to perform genomic engineering (Dahmann 2008; Venken and Bellen 2005).

As a consequence, the operators of a modern fly laboratory have to deal not only with the specific body of knowledge about its respective research area and scientific questions but also to incorporate the enormous breadth of genetic tools and strategies available in the fly, ranging from Mendelian rules and their *Drosophila*-specific aspects to sophisticated recombination-based strategies for the generation of mutant alleles or mosaic tissues. Accordingly, genetics training is a key requirement in *Drosophila* research groups. All those joining the laboratory without fly experience, including postdocs, PhD students, technical staff, and undergraduate project students, have to go through the bottleneck of the initial training that provides them with sufficient understanding of *Drosophila* genetics and the breadth of genetic tools that are in daily use in most fly laboratories. Only through this training will they start functioning in their daily routine, achieve a certain level of independence in their experimental work, and be able to comprehend advanced information resources to develop a greater degree of sophistication. Training new laboratory members or project students in fly genetics is time-consuming and demanding for the trainer yet too important to be left to chance.

Considering the size of the *Drosophila* community and its long-standing history, there is surprisingly little training material publicly available. Currently, excellent books are on hand to help the partly trained fly researcher to advance to the next step of expert knowledge in fly handling and advanced genetics (Ashburner *et al.* 2005; Dahmann 2008; Greenspan 2004). However, these resources do not answer seemingly trivial questions that a complete novice is initially faced with, often concerning very basic aspects of Mendelian rules in the fly, genetic terminology and gene names, marker mutations and balancers, the various classes of transgenic flies, and the principal strategies for their generation. A thorough “starter package” that provides an overview of these fundamental aspects of fly genetics is currently not available.

Here we present such a training package. It was developed over many years of teaching *Drosophila* mating schemes to undergraduate students and, since 2009, has been adapted and successfully established as a standard procedure to train new group members joining the Manchester fly facility. This package is composed of four individual modules, including self-study of an introductory manual, a short practical session on gender and marker selection, an interactive PowerPoint presentation, and finally independent training exercises in mating scheme design. Furthermore, a genotype builder is provided to help trainers generate fly images for their training tasks, useful also to illustrate presentations or publications.

## The Training Package

### Key rationale and flow chart of the training

Training newcomers to fly genetics is a far more complex task than is often appreciated by the experienced fly researcher. We may take it for granted that the importance of *Drosophila* is appreciated, that basic rules of Mendelian genetics are known to trainees, or that it is clear why we have to use mating schemes during our daily work. These suppositions often do not reflect reality. From our own experience, we identified a number of objectives that need to be achieved in basic fly genetics training:

- a basic appreciation of why the fly is being used for research;
- an understanding of why genetic crosses are required, what the nature of the involved fly stocks is (including classical alleles as well as transgenic fly lines), and why crosses need to be carefully planned through mating scheme design;
- an understanding of classical genetic rules, including the law of segregation, the law of independent assortment, and the nature of linkage groups and meiotic crossing-over—all in the context of *Drosophila* genetics;
- a knowledge of the nature and use of genetic markers and balancer chromosomes and how they are used for stock keeping and the unequivocal tracing of chromosomes within a mating scheme;
- an appreciation of genetic recombination as a threat but also experimental opportunity and how to manage recombination in mating schemes.

To meet these objectives we developed, as the first training module, a “Rough guide to *Drosophila* mating schemes” (Supporting Information, File S1). This manual is studied independently by trainees and prepares them for the rationale, rules, terminology, and genetic tools they are confronted with during the next steps of the training. Notably, it allows the trainees to ask informed questions and better engage in the further training process.

As the next step, we find it useful to provide the trainee with a brief practical training session on basic gender and marker selection. A simple training exercise (File S2) is provided that helps trainees to compare marker mutations of flies in reality with those used as images in the training modules. This short practical enhances the experience of the further training and makes it easier to link theory to practice.

A PowerPoint presentation is used as the next self-study module (File S3). It briefly reminds trainees of the key rules and facts before engaging in a detailed step-by-step guided tour through the design of a problem-driven mating scheme. This presentation demonstrates how the learned theory is applied during mating scheme design.

Up to this point, most training is performed through self-study and does not require more input than the brief training session in gender and marker selection and answering occasional queries. Therefore, these modules enormously reduce the time invested in training but also spare the trainees the need to ask naive or basic questions that may be perceived as embarrassing.

After this initial self-training, trainees are asked to solve genetic tasks reflecting simple day-to-day problems experienced in a fly laboratory (File S4). Trainees try to carry out this task independently but are encouraged to seek help in two ways. First, they should revisit the other training modules because information given in manual and presentation consolidates and becomes clearer when it is actively applied during mating scheme design. Second, trainees are invited to ask the trainer focused questions whenever necessary. Through this, trainees and teachers have a unique opportunity to identify which aspects of the training were well understood and where strengths and weaknesses lie for each individual trainee. These can then be tackled through discussion and reiteration of the learned material, thus achieving the basic training objective.

### The “rough guide to *Drosophila* mating schemes” (one day of trainee’s time)

The “rough guide to *Drosophila* mating schemes” (File S1) is handed out to trainees for initial self-study. It covers a wide range of topics, each based on a clear rationale.

The first section provides the key arguments for using flies and provides a brief historical perspective. For the young researcher, it is not necessarily clear why work on the fly may be important, especially in times when mouse or zebrafish genetics have become very powerful and where human disease has become a central topic in biomedical research. Understanding the roots of *Drosophila* genetics and how it contributed to subsequent scientific successes is key for appreciating the unbroken value of fly work in modern biology. References are provided to substantiate the arguments and encourage further reading.

The second section explains why genetic crosses are required and what the nature of the fly stocks is that are involved in these crosses. It introduces to the concepts of loss- and gain-of-function approaches and of forward and reverse genetics. Some of these aspects may seem trivial to the experienced drosophilist. Yet, their understanding is a fundamental prerequisite for novices to appreciate the importance of facts and rules learned in the following sections. If not explained, this poses a substantial barrier to understanding.

The third section addresses the practical aspects of handling flies in the laboratory. The intention is to raise awareness and introduce to good laboratory practice in fly handling but also to explain the manual work involved in the actual crosses behind mating schemes, thus pre-empting questions that will naturally arise during the next chapters of the manual.

**Figure 1:**
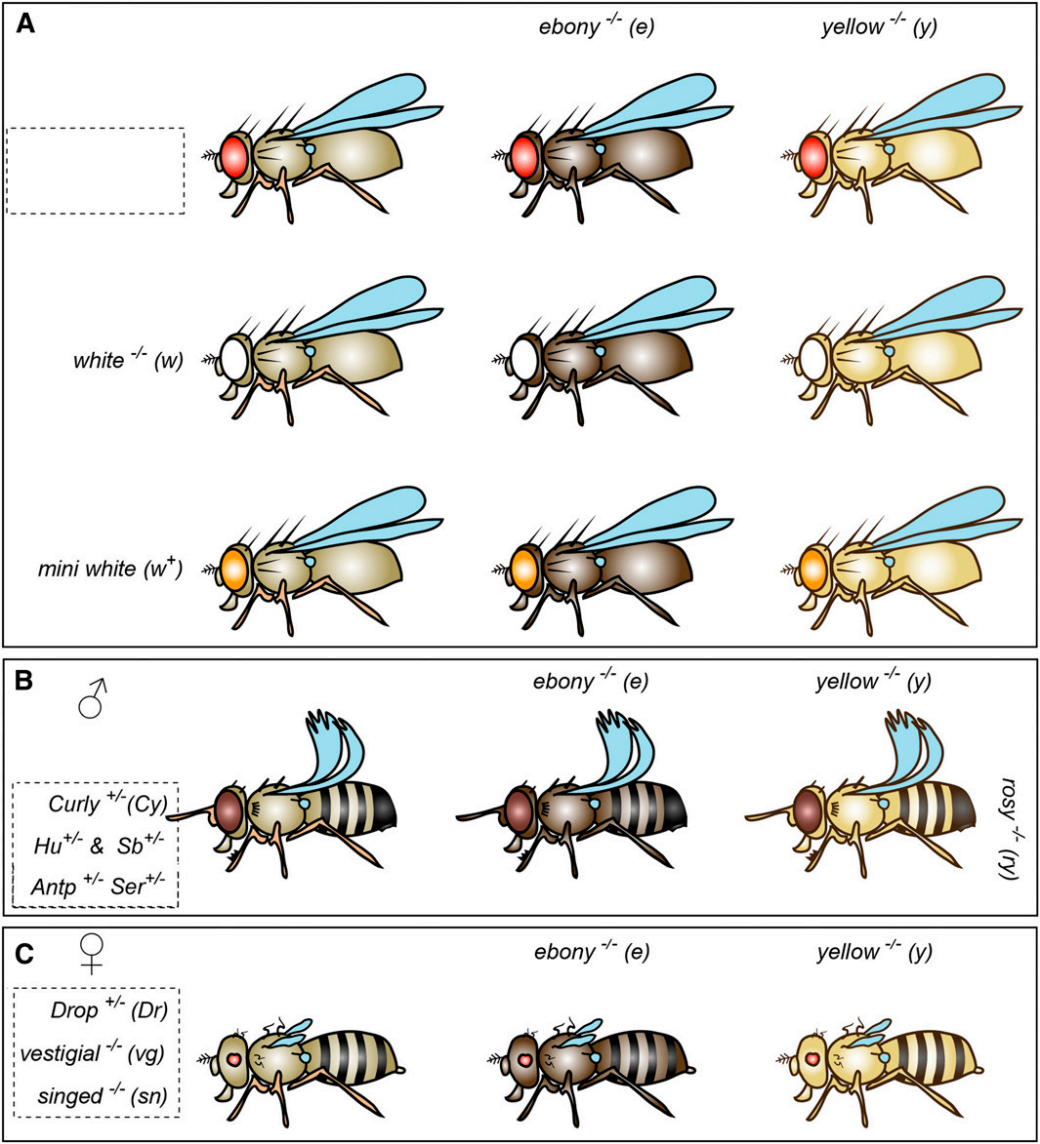
Simple and easy-to-grasp schematics illustrating common *Drosophila* marker mutations. All images were generated with the “Genotype Builder” Photoshop file (File S5). (A) The default set of flies (bristle, wing and eye markers set to “wildtype”) displays wild-type body color (left column), ebony (middle column), and yellow (right column) and normal eye color (top row), white mutant eyes (middle row), and orange eyes (mini-white or *w*^*apricot*^) (bottom row). (B) Example (top row only) with the settings “male” (fused abdominal stripe, sex combs, male anal plate), BRISTLES-Sb-Hu (Stubble_+/2_, short blunt bristles; Humeral_+/2_, multiplied humeral bristles), “EYE-wt” (normal shaped eyes) combined with OTHER-ry (*rosy^−/−^*, brown eyes), “OTHER-Antp” (*Antennapedia^+/−^*; antenna-to-leg transformation typical of the *Antp^73b^* mutation), “WINGS-Cy-Ser” (*Curly^+/−^*, curly wing; *Ser^+/−^*, notched wing tips). (C) Example (top row only) with the settings “female” (nonfused abdominal stripes, little protrusions of anal plates), EYES-Dr (*Drop^+/−^*, severely reduced eyes), “BRISTLES-sn” (singed^−/−^; curled bristles) and WINGS-vg (*vestigial^−/−^*; severely reduced wings).

The fourth section introduces trainees to the design of mating schemes, including the classical genetic rules, marker mutations, and balancer chromosomes. In its first part, it covers the principles and *Drosophila*-specific aspects of the Mendelian law of segregation, the confusing area of mutant alleles and their terminology, the Mendelian law of independent assortment, and the concepts of linkage groups and recombination. Within this context, additional information is given, including strategies in how best to write down mating schemes, brief descriptions of *Drosophila* chromosomes, peculiarities of the 4th chromosome, the recombination rule, gene descriptors and locators including the naming of fly genes. After this, the nature and use of genetic markers and balancer chromosomes is explained, with an introduction to the fly schematics generated by the genotype builder (Figure 1). In general, the trainee is made aware of the genetic recombination phenomenon as a threat but also an experimental opportunity and how balancers and the recombination rule can be used to manage recombination during mating schemes. Given the complexity of all these topics, a number of figures and information boxes are used to illustrate the content and provide simple examples.

The fifth section covers transgenesis as an important pillar of fly research. The manual primarily uses *P*-elements to illustrate the construction of transgenic fly lines, including the molecular organization of *P*-element vectors, some key applications (trap screens, transposon-mediated mutagenesis, homologous recombination), and some of the technical problems (size limitations of inserts, position effects, insertion hot/cold spots) and how they can be overcome. The chapter continues by explaining different classes of transgenic flies, somatic recombination (including mosaic analysis with a repressible cell marker, *i.e.*, MARCM) and the generation of germline clones (together with the concept of maternal contribution). Other classes of transposons and recombinases, the concept of enhancer−promoter lines, and the large-scale projects aiming to saturate the fly genome with transposon insertions are briefly introduced to raise awareness, but references are given to encourage further reading. In conclusion, this chapter provides a short overview of key rationales and trends in modern fly genetics.

The manual concludes with a section on classical strategies for the mapping of mutant alleles or transgenic constructs, including simple crosses with multiple balancer lines, deletion and meiotic mapping, and some of the complications that may arise during complementation tests (*e.g.*, transvection, nested genes, noncoding RNA loci). This chapter contains a figure with detailed explanations of a simple mating scheme and illustrates how to apply the rules explained throughout the manual and terminates with a text box summarizing the basic rules for designing mating schemes.

In conclusion, “The rough guide to *Drosophila* mating schemes” provides an overview of the topics required to understand why and how genetics is being used in a fly laboratory. Throughout this document, references to more detailed publications and web-based resources are given to raise awareness, encourage further self-study and gradually introduce trainees to the use of these resources.

### A brief training session on genetic marker mutations and gender selection (~1 hr of both trainee’s and trainer’s time)

Throughout the introductory manual, images of flies carrying different marker mutations are being used. These schematics overemphasize the respective phenotypes which, in our experience, is ideal for theoretical training purposes and preferable to using photos of real flies displaying markers mutations. To give trainees a realistic impression of these marker mutations, we routinely perform a short training exercise. For this, the trainees inspect flies carrying five different marker combinations (two flies per genotype) under a dissection microscope. Their phenotypes have to be identified among a group of 10 fly images on a parallel handout (File S2). In addition, the 10 images have to be assigned to 10 corresponding genotypes. Afterward, trainees are asked to sort the flies by gender. To reduce preparation time, we preselect flies of the five different genotypes and sort them into centrifuge tubes which can be stored in the freezer for many months and used as required. To enable teachers and trainers to generate new illustrations for their individual tastes and needs, the Genotype Builder is provided. It is an easy-to-use Photoshop file to generate images of flies carrying arbitrary combinations of common eye, wing and bristle markers (Figure 1; File S5).

### A PowerPoint presentation demonstrating how rules are applied (1-2 hr of trainee’s time)

A PowerPoint presentation (File S3) is used to demonstrate how the knowledge acquired during the first two modules is applied during mating scheme design. First, the presentation briefly reiterates the principal features of meiosis *vs.* mitosis and the key rules of fly genetics. Then, a standard laboratory task is described in which a homozygous viable *P*-element insertion on the second chromosome has to be recombined with a second chromosomal, recessive, homozygous lethal mutation. To perform this task, two separate stocks carrying the mutation and the *P*-element insertion, respectively, and a third fly stock with different balancer chromosomes are given. The presentation leads through the solution of this task step by step, illustrating and explaining the various strategic considerations and decisions that have to be taken and how the rules of *Drosophila* genetics are applied. At each step of the mating scheme trainees are prompted to make their own suggestions before they are presented with a possible solution. The presentation includes an example of a dead-end solution, demonstrating how trial and error and creative and flexible solution seeking usually lead to optimal cross design.

### Solving of crossing tasks through mating scheme design (~1 d of trainee’s, ~2 hr of trainer’s time)

A number of genetic tasks (File S4), comparable with the ones in the PowerPoint presentation, are handed out to the trainees, and they are asked to design suitable mating schemes. Trainees are encouraged to perform this exercise as independently as possible, extensively using the introductory manual and presentation as a source of help. In our experience, this is the training stage at which learned information manifests as true knowledge. Through revisiting the “Rough guide” and PowerPoint presentation having clear questions in mind, these training materials tend to be far better understood by trainees. Importantly, trainees are not left alone in this process, but strongly encouraged to come forward with concrete questions when problems arise. The questions asked by the trainees provide valuable insights for the teacher/trainer to pinpoint the individual gaps in understanding and correct for these through discussion and reiteration of learned material in a personalized manner.

## Discussion

The training package we have introduced here attempts to present, in a logical sequence, the aspects of *Drosophila* genetics that are essential for a newcomer to fly research. It does not attempt to go beyond the level that a new fly pusher would reach in due course. However, this package is designed to speed the learning process and ensure that there are no gaps in the students’ basic knowledge and understanding. The aim is to overcome initial problems by efficiently providing training on key aspects of classical and modern fly genetics at the very beginning of a candidate’s fly training. The package combines different didactic strategies to meet individual needs and helps to consolidate the learned information. As an essential benefit, it takes a huge burden off the trainer’s shoulders because essential parts are based on self-study. Furthermore, through the genetic task exercise it provides an effective and objective means to assess the training success and individualize training where required.

We have taken care that the training package raises awareness of important resources including FlyBase, stock centers, advanced literature, and other online aids. For example, by providing various links to FlyBase or Bloomington from different sections of the introductory manual, the training package familiarizes the student with valuable online resources and illustrates how to use them. As another example, the training package takes care to introduce trainees to historical roots and the importance of *Drosophila*, again pointing out helpful references. We believe it is important to understand how *Drosophila*’s successful use as a “boundary object” that can bridge the gap between genetics (as a tool) and other biological disciplines (as the task) (Keller 1996), has helped in the past to unravel fundamental principles of complex phenomena such as development (Lawrence 1992), signaling pathways (Cadigan and Peifer 2009), cell-cycle regulation (Lee and Orr-Weaver 2003), or nervous system functions (Bellen *et al.* 2010; Weiner 1999). Given the speed at which disease-relevant human genes are currently being discovered, the demand for tractable model organisms that can unravel the principal functions behind these genes is likely to increase. Functions of such genes will undoubtedly touch on complex areas such as lipid metabolism (Kühnlein 2011), glycosylation (Fabini and Wilson 2001; Nishihara 2010), extracellular matrix (Broadie *et al.* 2011), endocytic trafficking (Gonzalez-Gaitan 2003), cytoskeleton (Sánchez-Soriano *et al.* 2007), or chromatin regulation (Lyko *et al.* 2006) that seem ideal targets for systematic dissection through the fly.

To play this role well, appreciation of the importance of both classical and modern elements of fly genetics is pivotal, and we hope that our package helps to raise this awareness already at the early stages of *Drosophila* training. Maintaining the links to the classical body of knowledge is crucial and certainly considered an important remit of FlyBase and the fly stock centers. Excellent examples are the incorporation of Lindsley and Zimm (1992) into FlyBase, the impressive compendium by M. Ashburner (Ashburner *et al.* 2005), Fly Pushing (Greenspan 2004), or the fact that valuable genetic fly strains have been kept alive for many decades in public stock collections. This is important because many of the classical genetic problems and discoveries are being revisited in modern research and studied at the molecular level. For example, the classical models of homeotic gene complexes find their explanations in insulator elements and noncoding RNAs (Gummalla *et al.* 2012), classical knowledge on nondisjunction resurfaces in studies of meiosis (Spieler 1963), the vast knowledge on variegation and transvection can contribute to work on chromatin regulation (epigenetics) (Duncan 2002), or the importance of genome standardization for early genetic work (Kohler 1994) resurfaces as an issue in the context of high-throughput sequencing (Blumenstiel *et al.* 2009). Modern work can enormously benefit from the classical body of knowledge and the genetic tools generated many decades ago. However, to maintain these links, the fly community is faced with two challenges. First, classical journal and book publications can be difficult to obtain and should be made accessible in dedicated online libraries. Second, the genetic terminology and methods of communication in that literature slowly become incomprehensible for modern biologists. Easy training resources that prepare for the use of classical literature would therefore be invaluable.

The training package presented here could serve as a model for such initiatives not only for training in classical genetics but also for other aspects, such as a guide to *Drosophila*-relevant data bases and data mining strategies. Apart from such training packages, it is surprising that other simple and obvious aids seem to have never been developed by the fly community. One example for such an aid is the genotype builder introduced here as a quick and easy way to generate images of flies to illustrate genetic mating schemes for training or publication purposes. Furthermore, no software or online programs seem publicly available that would allow the easy formulation of genetic mating schemes so that they can be exchanged in digital format or used in publications. Certainly, the use of common word processing, illustration or presentation software is an unsatisfactory solution to this end. As another example, purpose-tailored database programs for *Drosophila* stocks do not exist. Such databases could be designed to link out to FlyBase (thus helping to raise standards of good practice in the use of nomenclature and stock documentation) and facilitate stock sharing between groups. Another action through which the fly community could facilitate daily life would be to widen the presence of *Drosophila* topics in Wikipedia or even develop a dedicated FlyWiki. Frequent comments we got from colleagues on our training material concerned the need to expand on the explanation of terms or concepts. While this would go far beyond the remit of our “Rough guide,” linking out to an online Wiki would provide an efficient means to extend to the next level of complexity in a flexible and individualized manner.

Finally, this training package originated from teaching *Drosophila* genetics to second-year undergraduate students, and most modules introduced here have already been used for student teaching. Teaching *Drosophila* mating schemes provides an excellent means to enrich the experience of students on practical courses and fill the time gaps between experimental steps of a protocol. Even more, it is an excellent way to excite students about genetics and show them how it can be used as a strategy to address fundamental questions of biology. The “rough guide” as introduced here certainly contains information that goes beyond basic teaching at the undergraduate level. However, a simpler version together with the other modules can provide a perfectly feasible training resource also for student courses. Along these lines, the scope of teaching about *Drosophila* can be taken a level further by having a more prominent presence on school curricula or on public outreach events. Increasingly, examples of such activities are being published (Harrison *et al.* 2011; Pulver *et al.* 2011), and aspects of the training material published here could provide further useful ideas for content and illustration for school teaching or outreach activities.

## Supplementary Material

Supporting Information
