# Supplementary material for: How to Design a Genetic Mating Scheme: A Basic Training Package for Drosophila Genetics
Source: G3 (Bethesda). 2013 Feb 1;3(2):353–8. doi: 10.1534/g3.112.004820 (PMC3564995; doi:10.1534/g3.112.004820)
Supplement: Supporting Information [file supp_3_2_353__index.html]

Supporting Information 

# How to Design a Genetic Mating Scheme: A Basic Training Package for *Drosophila* Genetics

## Supporting Information for Roote and Prokop, 2013

**Files in this Data Supplement:**

- Supporting Information - Supporting information available at  http://dx.doi.org/10.6084/m9.figshare.106631
