# Supplementary material for: How to Design a Genetic Mating Scheme: A Basic Training Package for Drosophila Genetics
Source: G3 (Bethesda). 2013 Feb 1;3(2):353–8. doi: 10.1534/g3.112.004820 (PMC3564995; doi:10.1534/g3.112.004820)
Supplement: Supporting Information [file supp_3.2.353_004820SI.pdf]

G3 Genes | Genomes | Genetics

# How to design a genetic mating scheme: a basic training package for Drosophila genetics

John Roote* and Andreas Prokop §

*) Faculty of Life Sciences, Wellcome Trust Centre for Cell-Matrix Research, Michael Smith Building, Oxford Road, Manchester M13 9PT, United Kingdom
§) Department of Genetics, University of Cambridge, Downing Street, Cambridge CB2 3EH, United Kingdom

10.1534/g3.112.004820

**Supporting Material**

The different training resources are described in detail in the text and are available under www.prokop.co.uk/XYZ.zip or at http://www.g3journal.org/lookup/suppl/doi:10.1534/g3.112.004820/-/DC1.

**File S1** PDF with the introductory manual "Rough guide to Drosophila mating schemes"
**File S2** PDF with the handout for the genetic marker training session
**File S3** PowerPoint presentation covering brief reminder of key rules and an example of a mating scheme.
**File S4** PDF containing some examples of training crossing tasks
**File S5** Adobe Photoshop file with the "Genotype Builder". Follow these instructions:

1. Open in Photoshop
2. Open "Window/Layers" from the menu bar
3. De-select the top layer "Instructions"
4. Select exactly one "GENDER", "EYE", "WINGS" and "BRISTLES" layer, respectively
5. Items entitled "OTHERS" provide further options that can be selected in addition; note that "OTHERS-ry" has to be used in combination with "EYE-wt"
6. Either print directly or select "Layer/Flatten image" option from the menu bar, save under a different name (preferably as a JPEG file) and cut out the fly with the correct body and eye colour
